# Metabolomics in psoriatic disease: pilot study reveals metabolite differences in psoriasis and psoriatic arthritis

**DOI:** 10.12688/f1000research.4709.1

**Published:** 2014-10-21

**Authors:** April W. Armstrong, Julie Wu, Mary Ann Johnson, Dmitry Grapov, Baktazh Azizi, Jaskaran Dhillon, Oliver Fiehn

**Affiliations:** 1 Department of Dermatology, University of Colorado Denver, Aurora, CO, 12801, USA; 2 Department of Dermatology, University of California Davis, Sacramento, CA, 95816, USA; 3 NIH West Coast Metabolomics Center, University of California Davis, Davis, CA, 95616, USA

## Abstract

**Importance:** While “omics” studies have advanced our understanding of inflammatory skin diseases, metabolomics is mostly an unexplored field in dermatology.

**Objective:** We sought to elucidate the pathogenesis of psoriatic diseases by determining the differences in metabolomic profiles among psoriasis patients with or without psoriatic arthritis and healthy controls.

**Design:** We employed a global metabolomics approach to compare circulating metabolites from patients with psoriasis, psoriasis and psoriatic arthritis, and healthy controls.

**Setting:** Study participants were recruited from the general community and from the Psoriasis Clinic at the University of California Davis in United States.

**Participants:** We examined metabolomic profiles using blood serum samples from 30 patients age and gender matched into three groups: 10 patients with psoriasis, 10 patients with psoriasis and psoriatic arthritis and 10 control participants.

**Main outcome(s) and measures(s):** Metabolite levels were measured calculating the mean peak intensities from gas chromatography time-of-flight mass spectrometry.

**Results:** Multivariate analyses of metabolomics profiles revealed altered serum metabolites among the study population. Compared to control patients, psoriasis patients had a higher level of alpha ketoglutaric acid (Pso: 288 ± 88; Control: 209 ± 69; p=0.03), a lower level of asparagine (Pso: 5460 ± 980; Control: 7260 ± 2100; p=0.02), and a lower level of glutamine (Pso: 86000 ± 20000; Control: 111000 ± 27000; p=0.02). Compared to control patients, patients with psoriasis and psoriatic arthritis had increased levels of glucuronic acid (Pso + PsA: 638 ± 250; Control: 347 ± 61; p=0.001). Compared to patients with psoriasis alone, patients with both psoriasis and psoriatic arthritis had a decreased level of alpha ketoglutaric acid (Pso + PsA: 186 ± 80; Pso: 288 ± 88; p=0.02) and an increased level of lignoceric acid (Pso + PsA: 442 ± 280; Pso: 214 ± 64; p=0.02).

**Conclusions and relevance:** The metabolite differences help elucidate the pathogenesis of psoriasis and psoriatic arthritis and they may provide insights for therapeutic development.

## Introduction

Psoriasis is a chronic, inflammatory skin disease associated with significant morbidity and mortality ^1^. Approximately one-third of psoriasis patients develop psoriatic arthritis ^2, 3^. The exact mechanisms underlying psoriasis remain under investigation. A complex interplay between genetic and environmental factors initiates a cascade of events that lead to activation of dendritic cells ^4^. The activated dendritic cells stimulate differentiation and migration of effector T cells (Th1 and Th17) to the skin. The subsequent release of inflammatory cytokines promotes further recruitment of immune cells, stimulates keratinocyte proliferation and sustains a state of chronic inflammation ^4^.

Genomics, proteomics, and transcriptomics research has substantially contributed to the growing body of knowledge concerning psoriasis etiology. For example, genome-wide transcriptional analysis and association studies have identified new susceptibility loci that implicate pathways in psoriasis pathogenesis which integrate epidermal barrier dysfunction with immune dysregulation ^5– 7^. A recent proteomic analysis of psoriatic skin tissue identified 36 up-regulated proteins associated with the regulation of cell death, defense response, inflammatory response, and reactive oxygen species ^8^. However, the data beyond genomics and proteomics at the metabolic level can provide new insights into the cellular processes involved in psoriasis.

Metabolomics, the most recent addition to the “omics” fields, is the comprehensive study of all low molecular mass metabolites in the metabolome of an organism under a given set of conditions ^9^. Examining the metabolic byproducts produced at the transcriptional and translational levels will yield clues to the cellular regulatory processes and underlying molecular networks. Because metabolites are the end products and the most downstream representation of cellular processes, the study of an organism’s metabolome is most reflective of any observable phenotype ^9^. Metabolomics analysis will enable researchers to gain valuable information regarding the physiology of a system by measuring the amplified output that results from genetic and environmental perturbations ^10^. To date, metabolomics has not yet been applied to investigate the systemic mechanisms underlying psoriasis.

Because psoriasis is a multifactorial disease at the genomic, protein, and cellular levels, it is important to examine the end products of cellular processes in psoriasis and psoriatic arthritis. In this novel study, we employed a global metabolomics approach using gas chromatography time-of-flight mass spectrometry (GC-TOF-MS) to compare the serum metabolic profiles of patients with psoriasis, patients with psoriasis and psoriatic arthritis, and healthy controls. We then summarized our results within the context of existing literature to generate hypotheses for future targeted analysis. Integrating genomics, proteomics, transcriptomics and metabolomics data from all levels will add to our understanding of the physiological processes underlying this complex disease and will provide potential insights into its associated comorbidities.

## Methods

### Study design

In this study, we examined the metabolite profiles of psoriasis patients with and without psoriatic arthritis and healthy controls using GC-TOF-MS. Blood serum samples were obtained from 30 patients: 10 patients with psoriasis, 10 patients with both psoriasis and psoriatic arthritis, and 10 healthy control participants. Patient demographics and treatments are summarized in Table 1. Plaque psoriasis is the most common subtype of psoriasis, and the diagnosis made by dermatologists based on characteristic red, scaly patches and plaques on characteristic locations. The Psoriasis Area and Severity Index (PASI) is a validated and widely used outcomes instrument in clinical trials that measures psoriasis severity. Psoriatic arthritis is an inflammatory arthritis that typically affects the peripheral joints but can also have axial manifestations, enthethitis, and dactylitis. In approximately 70–80% of patients with psoriatic arthritis, psoriasis precedes the development of joint symptoms. This study was approved by the Institutional Review Board at University of California, Davis (Protocol 254745-11). The Declaration of Helsinki was followed and the study participants provided their written, informed consent.

**Table 1.** Patient demographics. This table describes demographics and clinical information on the patient cohort that contributed their samples to this metabolomics study. Patient demographics indicated for patients with psoriasis (Pso), and patients with psoriasis and psoriatic Arthritis (PsA): age, sex, Body Mass Index (BMI), Psoriasis Area and Severity Index (PASI), duration of condition (in years), and current treatment(s). Demographics of age and sex for control patients are also indicated.

Psoriasis patients						Psoriasis and PsA patients						Control patients	
Age	Sex	BMI	PASI	Duration Pso (yrs)	Current treatment(s)	Age	Sex	BMI	PASI	Duration Pso/PsA (yrs)	Current treatment(s)	Age	Sex
60s	M	22.0	23.5	44	Acitretin 25mg qd, calcipotriene/ betamethasone dipropionate as needed, fluocinolone cream as needed	60s	M	31.3	7.2	Pso: 22 PsA: 22	Adalimumab 40mg every other week, clobetasol ointment 0.05% as needed, calcitriol as needed	60s	M
30s	M	25.8	14.8	11	Ustekinumab 45mg every 12 weeks, clobetasol cream 0.05% as needed	30s	M	37.1	21.4	Pso: 15 PsA: 12	Adalimumab 40mg every week, methotrexate 10mg weekly, calcipotriene as needed, fluocinonide cream as needed	30s	M
20s	F	28.8	12.2	13	Betamethasone diproprionate/ calcipotriene combination every day	40s	F	28.3	16.2	Pso: 1 PsA: 1	NSAIDs, Clobetasol ointment twice daily, triamcinolone as needed to the intertriginous areas	20s	F
30s	M	43.6	9.6	21	Triamcinolone 0.1% cream twice daily	20s	M	34.9	6.9	Pso: 4 PsA: 4	Adalimumab 40mg every other week, clobetasol cream 0.05% as needed, calcipotriene twice daily, hydrocortisone 2.5% cream as needed	30s	M
50s	M	28.4	9.6	17	Clobetasol ointment 0.05% daily, calcipotriene daily, etanercept 50mg every week	50s	M	26.3	2.6	Pso: 43 PsA: unknown	Methotrexate 15mg weekly, clobetasol cream 0.05% as needed, phototherapy (UVB) twice weekly	50s	M
40s	M	34.5	8	12	Clobetasol 0.05% cream as needed, calcipotriene as needed	40s	M	28.0	22.5	Pso: 35 PsA: 2	Etancercept 50mg weekly, betamethasone dipropionate cream as needed, coal tar as needed	50s	M
50s	F	23.0	6	45	triamcinolone cream 0.5% twice daily	50s	F	29.2	6.8	Pso: 11 PsA: 9	Etanercept 50mg every week, clobetasol ointment 0.05% as needed	60s	F
50s	F	28.3	5.2	4	Adalimumab 40mg every other week, halobetasol cream 0.05% as needed	50s	F	28.3	27.5	Pso: 6 PsA: 5	NSAIDs, methotrexate 12.5mg every week, clobetasol ointment 0.05% as needed, calcipotriene as needed	50s	F
40s	F	22.1	4.4	9	Clobetasol cream 0.05% as needed	40s	F	29.3	11.9	Unknown	NSAIDs as needed	40s	F
60s	F	32.6	3.2	7	Etanercept 50mg every week, clobetasol, calcipotriene/ betamethasone dipropionate as needed	70s	F	32.2	9	Unknown	NSAIDs as needed, fluocinonide cream as needed	70s	F

### Sample storage and preparation

Blood samples were collected from patients using a BD 5 ml double-gel serum separator tube. After 30 minutes of coagulation, the samples were immediately centrifuged at 2000 rpm for 15 minutes to complete blood fractionation ^11^. Thereafter, the sera were transferred to Eppendorf tubes, snap-frozen in liquid nitrogen for 60 s, and stored at -80°C until further processing.

### GC-TOF

Samples were analyzed using a GC-TOF approach. The study design was entered into the MiniX database ^12^. A Gerstel MPS2 automatic liner exchange system (ALEX) was used to eliminate cross-contamination from sample matrix occurring between sample runs. 0.5 microliter of sample was injected at 50°C (ramped to 250°C) in splitless mode with a 25 sec splitless time. An Agilent 6890 gas chromatograph (Santa Clara, CA) was used with a 30 m long, 0.25 mm i.d. Rtx5Sil-MS column with 0.25 µm 5% diphenyl film; an additional 10 m integrated guard column was used (Restek, Bellefonte PA) ^13– 15^. Chromatography was performed at a constant flow of 1 ml/min, ramping the oven temperature from 50°C for to 330°C over 22 min. Mass spectrometry used a Leco Pegasus IV time of flight mass (TOF) spectrometer with 280°C transfer line temperature, electron ionization at −70 V and an ion source temperature of 250°C. Mass spectra were acquired from *m/z* 85–500 at 17 spectra/sec and 1750 V detector voltage.

Result files were exported to our servers and further processed by our metabolomics BinBase database. All database entries in BinBase were matched against the Fiehn mass spectral library of 1,200 authentic metabolite spectra using retention index and mass spectrum information or the NIST11 commercial library. Identified metabolites were reported if present in at least 50% of the samples per study design group (as defined in the MiniX database); output results were exported to the BinBase database and filtered by multiple parameters to exclude noisy or inconsistent peaks ^15^. Quantification was reported as peak height using the unique ion as default. Missing values were replaced using the raw data netCDF files from the quantification ion traces at the target retention times, subtracting local background noise ^12^. Sample-wise metabolite intensities were normalized by the total signal for all annotated analytes. Daily quality controls and standard plasma obtained from NIST were used to monitor instrument performance over the length of the data acquisition.

### Data analysis

Statistical analysis was implemented on log2 transformed metabolite values in R ^16^. The significance levels (i.e. p-values) were adjusted for multiple hypothesis testing according to Benjamini and Hochberg ^17^ at a false discovery rate (FDR) of 5%.

Orthogonal signal correction partial least squares discriminant analysis (O-PLS-DA) ^18^ was carried in out in R ^16^ using the Devium package ( https://github.com/dgrapov/devium).

A biochemical and chemical similarity network ^19^ was developed for all measured metabolites with KEGG ^20^ and PubChem CIDs ^21^ identifiers. Enzymatic interactions were determined based on product-precursor relationships defined in the KEGG RPAIR database. Molecules not directly participating in biochemical transformations, but sharing many structural properties, based on PubChem Substructure Fingerprints ^22^, were connected at a threshold of Tanimoto similarity ≥ 0.7.

## Results

### Psoriasis patients compared to control participants

**Figure 1.**
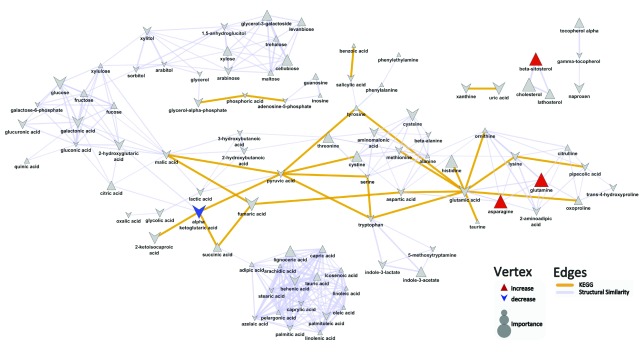
Biochemical similarity network analysis for metabolite differences in controls and psoriasis patients. Biochemical and chemical similarity networks were used to map statistical and multivariate modeling results within a biological context. The vertices represent metabolites which are connected by edges based on biochemical relationships (yellow, KEGG) and chemical similarities (lavender, structural similarity). Vertex size represents the metabolite's importance and the color represents the direction of statistically significant changes (blue, decrease; red, increase; gray, insignificant p>0.05).

Analysis of the human sera samples using GC-TOF-MS yielded 144 known metabolic structures and a total of 354 molecular features ( Dataset). When comparing the metabolite profiles of psoriasis patients and control patients, the key differences that reached univariate significance were characterized by an increase in alpha ketoglutaric acid (mean peak intensity ± std dev: Pso: 288±88; Control: 209±69; p=0.03), and decreases in asparagine (mean peak intensity ± std dev: Pso: 5460±980; Control: 7260±2100; p=0.02), glutamine (mean peak intensity ± std dev: Pso: 86000±20000; Control: 111000±27000; p=0.02), and beta-sitosterol (mean peak intensity ± std dev: Pso: 217±100; Control: 315±130; p=0.04) in psoriasis patients compared to control patients ( Figure 1, Dataset). OPLS-DA showed that all models performed significantly better (Q ^2^=0.42±0.1; RMSEP=0.37±0.1) than what is expected based on random chance (permuted: Q ^2^=-0.51±0.7; RMSEP=0.68±0.1).

### Patients with psoriasis and psoriatic arthritis compared to patients with psoriasis

**Figure 2.**
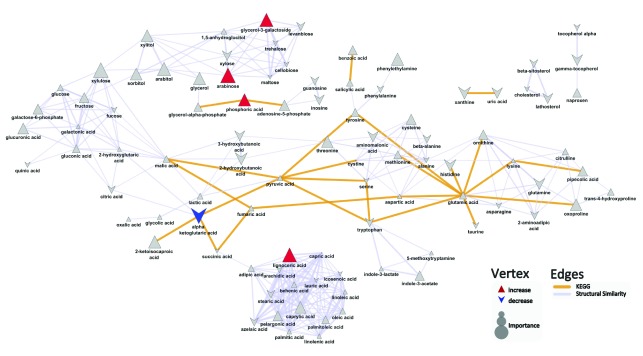
Biochemical similarity network analysis for metabolite differences between psoriasis patients with and without arthritis. Biochemical and chemical similarity networks were used to map statistical and multivariate modeling results within a biological context. The vertices represent metabolites which are connected by edges based on biochemical relationships (yellow, KEGG) and chemical similarities (lavender, structural similarity). Vertex size represents the metabolite's importance and the color represents the direction of statistically significant changes (blue, decrease; red, increase; gray, insignificant p>0.05)

Upon comparing the metabolite profiles of patients with both psoriasis and psoriatic arthritis to patients with skin-limited psoriasis, the top metabolite differences were characterized by a decrease in alpha ketoglutaric acid (mean peak intensity ± std dev: Pso+PsA: 186±80; Pso: 288±88; p=0.02), and increases in arabinose (mean peak intensity ± std dev: Pso+PsA: 624±460; Pso: 363±90; p=0.04), lignoceric acid (mean peak intensity ± std dev: Pso+PsA: 442±280; Pso: 214±64; p=0.02), phosphoric acid (mean peak intensity ± std dev: Pso+PsA: 20600±4300; Pso: 17100±3300; p=0.05), and glycerol-3-galactoside (mean peak intensity ± std dev: Pso: 193±140; Pso+PsA: 367±190; p=0.02) in psoriasis patients with concomitant psoriatic arthritis compared to patients with psoriasis alone ( Figure 2, Dataset). The OPLS-DA results suggest that the developed model did not perform significantly better (Q ^2^=0.66±0.2; RMSEP=0.54±0.1) than what is expected based on random chance (permuted: Q ^2^=0.47±0.3; RMSEP=0.55±0.1).

### Patients with psoriasis and psoriatic arthritis compared to control participants

**Figure 3.**
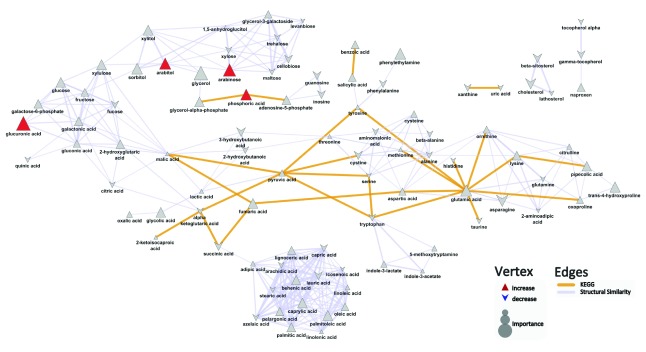
Biochemical similarity network analysis for psoriasis patients with psoriatic arthritis compared to controls. Biochemical and chemical similarity networks were used to map statistical and multivariate modeling results within a biological context. The vertices represent metabolites which are connected by edges based on biochemical relationships (yellow, KEGG) and chemical similarities (lavender, structural similarity). Vertex size represents the metabolite's importance and the color represents the direction of statistically significant changes (blue, decrease; red, increase; gray, insignificant p>0.05).

Key metabolite differences between patients with both psoriasis and psoriatic arthritis and healthy controls are characterized by increases in phosphoric acid (mean peak intensity ± std dev: Pso+PsA: 20600±4300; Control: 16300±4000; p=0.02), glucuronic acid (mean peak intensity ± std dev: Pso+PsA: 638±250; Control: 347±61; p=0.001), arabitol (mean peak intensity ± std dev: Pso+PsA: 238±65; Control: 172±61; p=0.03), and arabinose (mean peak intensity ± std dev: Pso+PsA: 624±460; Control: 322±140; p=0.02) in patients with both psoriasis and psoriatic arthritis compared to healthy individuals ( Figure 3, Dataset). OPLS-DA analysis suggested that the developed model performed better (Q ^2^=0.86±0.1; RMSEP=0.50±0.05) than what is expected based on random chance (permuted: Q ^2^=0.48±0.4; RMSEP=0.55±0.1) and displays weak predictive performance.

Data of metabolite differences in psoriasis and psoriatic arthritis A detailed legend of the raw data for Figures 1–3 can be found in the text file provided.

## Discussion

Psoriasis is widely recognized as a condition that is more than “skin deep”. The infiltration of inflammatory cells in psoriasis dermis and epidermis may be released into systemic circulation, contributing to chronic systemic inflammation ^23^. To our knowledge, this is the first study that employs a metabolomics approach to analyze human serum and examine metabolite changes in circulation beyond the skin. The spectral results indicated differences in low molecular weight compounds that may help distinguish psoriasis patients from healthy controls, psoriasis patients from patients with both psoriasis and psoriatic arthritis, and psoriasis patients with psoriatic arthritis from healthy controls. Although our metabolomics analysis detected a variety of altered metabolite levels, many of which warrant investigation, herein, we discuss the altered levels of the metabolites where the most relevant literature exists. We place our findings within the context of existing literature and explore their relationship to the immune-mediated and inflammatory nature of the disease.

### Psoriasis patients compared to control participants

***Glutamine.*** Glutamine, the most abundant free amino acid in the body, is essential for protein synthesis and cellular growth ^24^. In this study, GC-TOF-MS analysis revealed a decrease in glutamine levels in the sera of patients with psoriasis when compared to healthy controls. The alteration in glutamine levels may be due to higher rates of protein synthesis, glutamine consumption by immune cells, and/or differences in transglutaminase levels between patients with and without psoriasis.

Psoriasis is characterized by high rates of cellular proliferation, which increases the cellular demand of amino acids, notably glutamine, to accommodate for the higher rate of protein synthesis. This increased demand for glutamine is analogous to alterations of glutamine levels reported in patients with cancer, a condition also characterized by increased cellular proliferation ^24, 25^. In addition, psoriasis is also characterized by a dysregulated immune system whereby the amount and activity of immune cells are enhanced ^4^. The proliferation and functions of immune cells are highly dependent upon glutamine. High rates of glutamine consumption exhibited by lymphocytes, macrophages, and neutrophils have been reported in numerous studies *in vitro* ^26, 27^. Specifically, glutamine consumption enhances the production of many cytokines, and among them, tumor necrosis factor-α (TNF-α), interferon-γ (IFN-γ), interleukin-1β (IL-1β), and interleukin-6 (IL-6), all of which play an important role in psoriasis innate immunity.

Another possible explanation for the observed decrease in glutamine levels may be related to the up-regulation and increased expression of keratinocyte tranglutaminase in psoriatic lesions ^28, 29^. Keratinocyte transglutaminase is an enzyme critical for cornified envelope formation and participates in epidermal differentiation. Transglutaminase catalyzes the formation of the N-(γ-glutamyl)-lysine isopeptide bond using the amino acids glutamine and lysine. The inhibition of keratinocyte transglutaminase expression by retinoic acid may explain, in part, one of the beneficial effects of retinoid use to treat psoriasis.

***Asparagine.*** Asparagine, a common non-essential amino acid, is the amide of aspartic acid and is easily hydrolyzed during the cell aging process ^30, 31^. The spectral results showed a decrease in asparagine levels in the serum of psoriasis patients, which may result from spontaneous asparagine deamidation, a process enhanced by an oxidative microenvironment. Chronic oxidative stress has been reported in patients with psoriasis ^32^. In asparagine deamidation, a spontaneous post-biosynthetic modification, the asparagine residue undergoes nucleophilic attack and forms a succinimide ring intermediate. The succinimidyl residue is an unstable five-membered ring that hydrolyzes to give a mixture of aspartyl and isoaspartyl forms ^30^. The resulting isoaspartate residue (isoAsp) is formed through a beta linkage as opposed to the normal alpha conformation, and is favored 3:1 over the normal conformation at physiological conditions ^31^. The presence of isoAsp residues in proteins has been shown to decrease protein function and can trigger autoimmune responses ^31^. However, a protein repair system that employs the enzyme L-isoAsp-(D-Asp)-O-methyltransferase (PIMT) has recently been identified. PIMT specifically targets the isoAsp residues and forms an unstable methyl ester (L-isoaspartyl methyl ester), which is rapidly converted back to the L-succinimidyl form. The L-succinimidyl residues are then hydrolyzed to the L-aspartyl form ^30^. This repair system prevents the accumulation of damaged proteins.

The observed decrease in asparagine is consistent with the findings from a study by D’Angelo *et al.* (2012), who examined the abnormal isoaspartyl residues in the erythrocyte membranes of psoriasis patients ^33^. D’Angelo *et al.* found that L-isoAsp content was highly increased in the red blood cells (RBC) membrane proteins of psoriatic patients. By measuring methyl esterification through an *in situ* protein methylation approach, the investigators found that protein methyl esterification was increased by 1.5 fold in psoriatic patients compared to controls. To determine whether these data are due either to an increase in deamidation rate or to an impaired repair system in patients with psoriasis, they measured the intracellular levels of S-adenosylmethionine (SAM) and S-adenosylhomocysteine (SAH). D’Angelo *et al.* found that the transmethylation potential (SAM/SAH) was comparable in patients and controls, indicating a normal repair system in psoriasis patients. Therefore, the increase in methyl esterification is more likely to be attributed to increased protein instability at asparagine sites, which is responsible for asparagine degradation and IsoAsp accumulation in psoriasis patients. Of note, isoAsp residues may confer immunogenicity to previously ignored self-antigens and need to be further explored given the immune-mediated nature of psoriasis ^31^.

***Alpha ketoglutaric acid.*** Alpha ketoglutarate is an intermediate of the citric acid cycle involved in cellular metabolism. The increase in alpha ketoglutaric acid levels detected in serum of psoriasis patients may be due to enhanced alpha ketoglutarate synthesis. Alpha ketogluarate is synthesized from isocitrate through a process mediated by isocitrate dehydrogenase. Enzymatic assays from previous studies have reported significantly increased enzymatic activities of isocitrate dehydrogenase in psoriasis patients ^34^.

The increase in alpha ketoglutaric acid levels may be a contributing factor to the increase in cellular proliferation associated with psoriasis. Singh *et al.* (2013) and Vishnoi *et al.* (2013) found that the *in vitro* addition of alpha ketoglutarate greatly enhanced cellular proliferation rates, cell survival, and metabolic activity. Alpha ketoglutarate functions as a scavenger for removing toxic metabolites within cells, thereby enhancing cell viability. Additional studies support alpha ketoglutarate involvement in cellular proliferation through regulation of the mTOR-signaling pathway ^35^. mTOR, a protein kinase responsible for cell proliferation, cell survival, and protein synthesis, is phosphorylated by alpha ketoglutarate ^35^. Therefore, increased alpha ketoglutarate levels may also activate mTOR pathways and induce cellular hyperproliferation.

Altered alpha ketoglutaric acid levels may also play a role within the hyperactive immunogenic state of psoriasis, in which lymphocytes, macrophages, and neutrophils secrete the inflammatory cytokines TNF-α, IL-1, and IL-6. In cell cultures, alpha ketoglutarate appears to enhance macrophage cytotoxicity by significantly increasing TNF-α and nitric oxide release ^36^. Furthermore, TNF-α release from macrophages can activate collagen synthesis in dermal fibroblasts of psoriatic skin. Previous studies have observed that psoriasis is associated with increased collagen synthesis ^37– 39^. Collagen functions as an abundant connective tissue protein and provides structural integrity for the epidermis. During collagen synthesis, alpha ketoglutarate functions as a cofactor that activates Prolyl-4-Hydroxylase (P4H), an enzyme that mediates the post-translational modification of pre-procollagen peptides essential for formation of the collagen triple helix. Increased enzymatic activity of P4H enhances collagen synthesis in psoriatic lesions. Moreover, alpha ketoglutarate increases the available pool of proline residues required for collagen synthesis. Transamination of alpha ketoglutarate into glutamate, with subsequent conversion into proline, provides P4H with additional proline substrates to synthesize the hydroxyproline required in collagen formation ^37^. Taken together, higher concentrations of alpha ketoglutaric acid may contribute to the structural properties as well as the immune and inflammatory properties of psoriasis.

### Patients with psoriasis and psoriatic arthritis compared to patients with psoriasis

***Alpha ketoglutaric acid.*** As noted earlier, alpha ketoglutarate can act to facilitate collagen synthesis in psoriasis patients. Although psoriasis patients exhibit elevated alpha ketoglutarate levels compared to controls, patients diagnosed with psoriasis and psoriatic arthritis had lower serum alpha ketoglutarate levels. This may be the result of a higher inflammatory burden experienced by these patients. Inflammatory cytokines, particularly interleukins and TNF-α, are up-regulated in the synovial fluid of psoriatic arthritis patients, further enhancing inflammatory joint destruction and periarticular bone loss ^40^. Collagen functions as the primary structural component in cartilage, bones, and skin. Degradation of joint tissue in patients with psoriasis and psoriatic arthritis may significantly increase the demand for alpha ketoglutarate in an attempt to synthesize new collagen. This increased consumption may account for the decreased alpha ketoglutarate levels in patients with psoriasis and psoriatic arthritis.

***Lignoceric acid.*** Lignoceric acid, a saturated very long-chain fatty acid (VLCA), is a minor fatty acid component found in human tissues and the bloodstream. In this study, serum lignoceric acid levels were elevated in patients with psoriasis with both skin and joint involvement when compared to patients with skin-limited psoriasis. The levels of saturated VLCFA in erythrocytes, specifically lignoceric acid, have been used to evaluate atherogenicity in patients with metabolic syndrome ^41^. Similarly, Matsumori *et al.* (2013) found higher levels of lignoceric acid in the erythrocytes of patients with metabolic syndrome when compared to healthy controls ^41^. This increase significantly correlated with specific atherogenic lipoprotein profiles and systemic inflammation in patients with metabolic syndrome ^41^. A metabolic syndrome is characterized partly by insulin resistance resulting from chronically elevated levels of plasma free fatty acids that, when released into liver and muscle tissue, inhibit insulin secretion ^42^. Epidemiologic studies have shown an increased prevalence of metabolic syndrome in patients with psoriatic arthritis compared to patients with psoriasis only ^43^. As such, the increase in lignoceric acid levels is consistent with the notion that psoriasis patients afflicted with concomitant psoriatic arthritis may experience a greater inflammatory burden than patients with skin involvement alone.

### Patients with psoriasis and psoriatic arthritis compared to control participants

***Glucuronic acid.*** Glucuronic acid is one of the main subunits that comprise the backbone of glycosaminoglycans (GAGs). In the present study, levels of glucuronic acid were increased in patients with psoriasis and psoriatic arthritis when compared to healthy controls, which corroborates the role of GAGs in relation to psoriasis. GAGs are complex, negatively-charged polysaccharides that bind with proteins to form proteoglycans. These structures are intimately involved in crucial components of cell signaling, cell adhesion and cell migration as they modulate the activity of the proteins that they bind ^44^. Of note, GAGs have been proposed to store as well as activate growth factors in the extracellular matrix and cell surface ^44, 45^. This suggests that GAGs potentially play a significant role in influencing cell proliferation, differentiation, tissue remodeling, and regulation of extracellular matrix composition ^45– 49^. The GAGs present in the extracellular matrix of these cells may induce keratinocyte hyperproliferation and decrease epidermal differentiation in psoriasis.

Studies have shown elevated levels of GAGs, specifically chondroitin sulfate and dermatan sulfate, in dermatologic and urine samples of patients with psoriasis. Chondroitin sulfate is a class of GAG that consists of a disaccharide backbone composed of alternating D-glucuronic acid and N-acetyl-D-galatosamine, whereas dermatan sulfate is synthesized from the epimerization of the glucuronic acid residues to iduronic acid at the polymer level ^50^. Using antibody staining against chondroitin sulfate, Smetsers *et al.* (2004) found that the chondroitin sulfate was primarily expressed in the papillary dermis and the basal keratinocytes in normal skin samples, whereas there was a more diffuse staining extending into the reticular dermis in psoriatic skin samples ^51^. In a study examining the localization of various GAGs on skin cells of normal and psoriatic skin, Saga *et al.* (1995) found that psoriatic epidermis exhibited increased chondroitin sulfate as well as dermatan sulfate, verified through digestion by enzymes specific to certain GAGs ^52^. The researchers also noted a higher concentration of chondroitin sulfate and dermatan sulfate from the stratum basale to the lower stratum spinosum in psoriasis patients compared to patients with normal skin ^52^. Moreover, Poulsen *et al.* (1983) reported that the concentration of GAGs increased by 28% and 62% in the dermal and urine samples, respectively, of psoriatic patients versus the healthy controls ^53^. In another study, Poulsen *et al.* (1982) noted that the excretion of dermatan sulfate doubled to 104% (p < 0.01) and the excretion of chondroitin sulfate increased by 62% (p < 0.02) in psoriasis patients versus controls ^54^. However, only the excretion of dermatan sulfate correlated with the fraction of body surface area involved in the psoriasis patients ^54^. It has been suggested that the increased urinary excretion of GAGs indicates greater catabolism ^55^.

In patients with psoriasis and psoriatic arthritis, chronic inflammation often leads to irreversible joint destruction ^56^. Hyaluronan is another class of GAG composed of a D-glucuronic acid and N-acetyl-D-galatosamine backbone ^57^. As a major component of the extracellular matrix in the articular cartilage, the joint destruction and degradation of hyaluronan into smaller oligosaccharides and its main components may account for the increase in glucuronic acid levels detected in our study. Although literature is scarce regarding the levels of hyaluronan in psoriatic arthritis, studies have reported an increase in serum hyaluronan in patients with rheumatoid arthritis when compared to healthy individuals ^58– 61^. Of note, hyaluronan has exhibited a diverse biological role with opposing dual functions in the inflammatory process ^57^. Fragments of hyaluronan have been reported to enhance the inflammatory and catabolic response through the modulation of Toll-like receptor 2 signaling pathways ^62^. While the suppression of hyaluronan synthesis alleviates inflammatory responses in some arthritic models, hyaluronan can act as an inflammatory activator as well as an inflammatory moderator ^63, 64^. Taken together, because glucuronic acid is a crucial backbone of GAGs, these results are consistent with our findings of elevated glucuronic acid in psoriatic serum samples.

In this study, a global metabolomics approach allowed an unbiased analysis of the entire pool of low molecular weight metabolites. The metabolomics data generated using this approach must be integrated with prior knowledge to identify specific metabolites for targeted analysis in future studies and to validate/confirm findings. Our study has several limitations. The small sample size may be associated with the inability to detect potential differences in certain metabolites. In this pilot study, while we captured PASI scores, psoriasis patients were allowed to maintain their usual treatment regimens. The effect of different treatment agents on metabolite perturbations could not be determined in this pilot study. Future studies with a larger patient cohort are necessary to confirm these findings.

To our knowledge, this is the first study to evaluate the comprehensive metabolome of patients with psoriasis. Differences in the serum metabolites of psoriasis patients, psoriasis patients with psoriatic arthritis, and control patients were detected through GC-TOF-MS, and further studies with larger sample sizes are necessary to confirm our findings. The development of a well-characterized metabolomics profile for patients with psoriasis and psoriatic arthritis will contribute to understanding pathophysiology of psoriasis and its associated comorbidities. The metabolite differences between psoriasis patients and healthy individuals detected by global metabolomics analysis help elucidate the underlying mechanisms of psoriasis and provide the foundations for therapeutic development.

## Data availability

F1000Research: Dataset 1. Data of metabolite differences in psoriasis and psoriatic arthritis, 10.5256/f1000research.4709.d37090 ^65^

## Consent

All the participants provided a written informed consent to publish the data reported in this study. This study was approved by the Institutional Review Board at University of California, Davis.
