# Supplementary material for: Metabolomics in psoriatic disease: pilot study reveals metabolite differences in psoriasis and psoriatic arthritis
Source: F1000Res. 2014 Oct 21;3:248. [Version 1] doi: 10.12688/f1000research.4709.1 (PMC4288418; doi:10.12688/f1000research.4709.1)
Supplement: Peer review history

# Peer review: Referee response for version 1

**DOI:** 10.5256/f1000research.5031.r6927

**Published:** 2015-01-06

**Authors:** J Lee

**Affiliations:** 1 The Permanente Medical Group, TPMB, San Bruno, CA, USA

Interesting pilot study investigating metabolite differences between patients with psoriasis, those with psoriatic arthritis, and unaffected controls. I look forward to seeing these results replicated in a follow-up study which will include larger sample size. Controlling for treatment method, and comparison of patients who are on vs off treatment will also be necessary next steps. It will also be interesting to demonstrate in what manner such metabolites fluctuate with extent of disease. The mechanism behind these metabolite discrepancies and their significance in the disease pathogenesis also require further investigation. This study opens the door to multiple avenues of further study.

I have read this submission. I believe that I have an appropriate level of expertise to confirm that it is of an acceptable scientific standard.

# Peer review: Referee response for version 1

**DOI:** 10.5256/f1000research.5031.r6483

**Published:** 2014-11-10

**Authors:** Trilokraj Tejasvi

**Affiliations:** 1 Department of Dermatology, University of Michigan, Ann Arbor, MI, USA

This manuscript is very well written and informative. The shortcomings of this study are highlighted in the discussion; the sample size, the usage of medications and the exploratory design. Although the statistical analyses used are appropriate, presenting nominal p values and the corrected p values would add reality to this study.

I have read this submission. I believe that I have an appropriate level of expertise to confirm that it is of an acceptable scientific standard.
